# Reappraising cardiac function with myocardial contraction fraction: normal values, disease detection, and prognostication

**DOI:** 10.1093/ehjci/jeag019

**Published:** 2026-01-22

**Authors:** Hibba Kurdi, George Thornton, Hunain Shiwani, Jessica Artico, Aderonke Abiodun, Silvia Castelletti, Stefania Rosmini, Sabrina Nordin, Joao Augusto, Rebecca Kozor, Viviana Maestrini, Lamia Al Saikhan, Uzma Gul, George Joy, Rebecca Hughes, Anish Bhuva, Benjamin Meredith, Gabriella Captur, Marianna Fontanna, Derralynn Hughes, Peter Kellman, Alun D Hughes, Erik Schelbert, Charlotte H Manisty, Thomas A Treibel, James C Moon, Rhodri H Davies

**Affiliations:** University College London, 62 Huntley Street, London WC1E 6DD, United Kingdom; Barts Heart Centre, London, United Kingdom; University College London, 62 Huntley Street, London WC1E 6DD, United Kingdom; Barts Heart Centre, London, United Kingdom; University College London, 62 Huntley Street, London WC1E 6DD, United Kingdom; Barts Heart Centre, London, United Kingdom; University College London, 62 Huntley Street, London WC1E 6DD, United Kingdom; Barts Heart Centre, London, United Kingdom; University College London, 62 Huntley Street, London WC1E 6DD, United Kingdom; Barts Heart Centre, London, United Kingdom; University Hospital Santa Maria della Misericordia, Udine, Italy; Kings College London, London, United Kingdom; University of Glasgow and Queen Elizabeth University Hospital; University College London, 62 Huntley Street, London WC1E 6DD, United Kingdom; Royal North Shore Hospital and University of Sydney, Sydney, Australia; Department of Clinical, Internal, Anesthesiological and Cardiovascular Sciences, Sapienza University of Rome, Italy; Department of Cardiac Technology, College of Applied Medical Sciences, Imam Abdulrahman Bin Faisal University, Kingdom of Saudi Arabia; Barts Heart Centre, London, United Kingdom; University College London, 62 Huntley Street, London WC1E 6DD, United Kingdom; University College London, 62 Huntley Street, London WC1E 6DD, United Kingdom; Barts Heart Centre, London, United Kingdom; University College London, 62 Huntley Street, London WC1E 6DD, United Kingdom; Barts Heart Centre, London, United Kingdom; University College London, 62 Huntley Street, London WC1E 6DD, United Kingdom; Barts Heart Centre, London, United Kingdom; University College London, 62 Huntley Street, London WC1E 6DD, United Kingdom; The Royal Free Hospital, London, United Kingdom; The Royal Free Hospital, London, United Kingdom; University College London, 62 Huntley Street, London WC1E 6DD, United Kingdom; The Royal Free Hospital, London, United Kingdom; National Heart, Lung, and Blood Institute, National Institutes of Health Bethesda, USA; University College London, 62 Huntley Street, London WC1E 6DD, United Kingdom; University of Pittsburgh, PA, USA; University College London, 62 Huntley Street, London WC1E 6DD, United Kingdom; Barts Heart Centre, London, United Kingdom; University College London, 62 Huntley Street, London WC1E 6DD, United Kingdom; Barts Heart Centre, London, United Kingdom; University College London, 62 Huntley Street, London WC1E 6DD, United Kingdom; Barts Heart Centre, London, United Kingdom; University College London, 62 Huntley Street, London WC1E 6DD, United Kingdom

**Keywords:** left ventricular hypertrophy, left ventricular ejection fraction, myocardial contraction fraction, cardiovascular magnetic resonance imaging, heart failure, cardiovascular imaging

## Abstract

**Aims:**

Assessing cardiac function is critical for managing cardiovascular disease, guiding treatment, monitoring progression, and risk stratification. While left ventricular (LV) ejection fraction (LVEF) is firmly established, it has limitations. Myocardial contraction fraction (MCF)—the ratio of stroke volume to myocardial volume, is simple to compute without additional analysis and offers a promising alternative to LVEF.

**Methods and results:**

MCF was assessed across four datasets spanning healthy controls and chronic structural cardiac disease, with direct comparison to LVEF. Association between age, sex, and MCF were investigated in 3541 healthy subjects from the UK Biobank and sex-specific reference ranges derived. Several cohorts were recruited to investigate the discriminative power of MCF and LVEF between health and physiological adaption (*n* = 278 veteran athletes), pathological hypertrophy [hypertrophic cardiomyopathy, amyloid, Fabry, severe aortic stenosis (AS), and hypertension (HTN); *n* = 633], and dilatation [*n* = 103 dilated cardiomyopathy (DCM)]. Ability to track disease severity was assessed by looking at 41 558 subjects from the UK Biobank. Finally, prognostication was assessed on 1277 consecutive patients from an independent external dataset. All images were analysed using the same validated artificial intelligence algorithm. MCF varied with sex (mean MCF: 0.94 male; 1.1 female) but not age. Sex-specific reference ranges were established: [0.68–1.20] for male and [0.82–1.38] for female. MCF decreased in pathological disease (e.g. mean MCF: 0.72 HCM; 0.69 severe AS; 0.5 amyloid; 0.9 HTN) but there was no significant decrease in LVEF other than in amyloid (mean EF: 76% HCM; 64% severe AS; amyloid 56%; 65% HTN). Both MCF and ejection fraction (EF) decreased in DCM (EF 34%; MCF 0.58). MCF decreased with worsening HTN, whereas LVEF increased (*P* < 0.05). MCF had superior prognostic ability to LVEF (MCF vs. LVEF: HR = 0.772 vs. HR = 0.816; *χ*^2^ = 198 vs. *χ*^2^ = 151; *P* < 0.001).

**Conclusion:**

We established MCF reference ranges, showing superior performance for detecting early disease and tracking progression compared with LVEF. MCF offers enhanced prognostic utility, complementing established metrics of LV function.

## Graphical Abstract

**Graphical Abstract:**
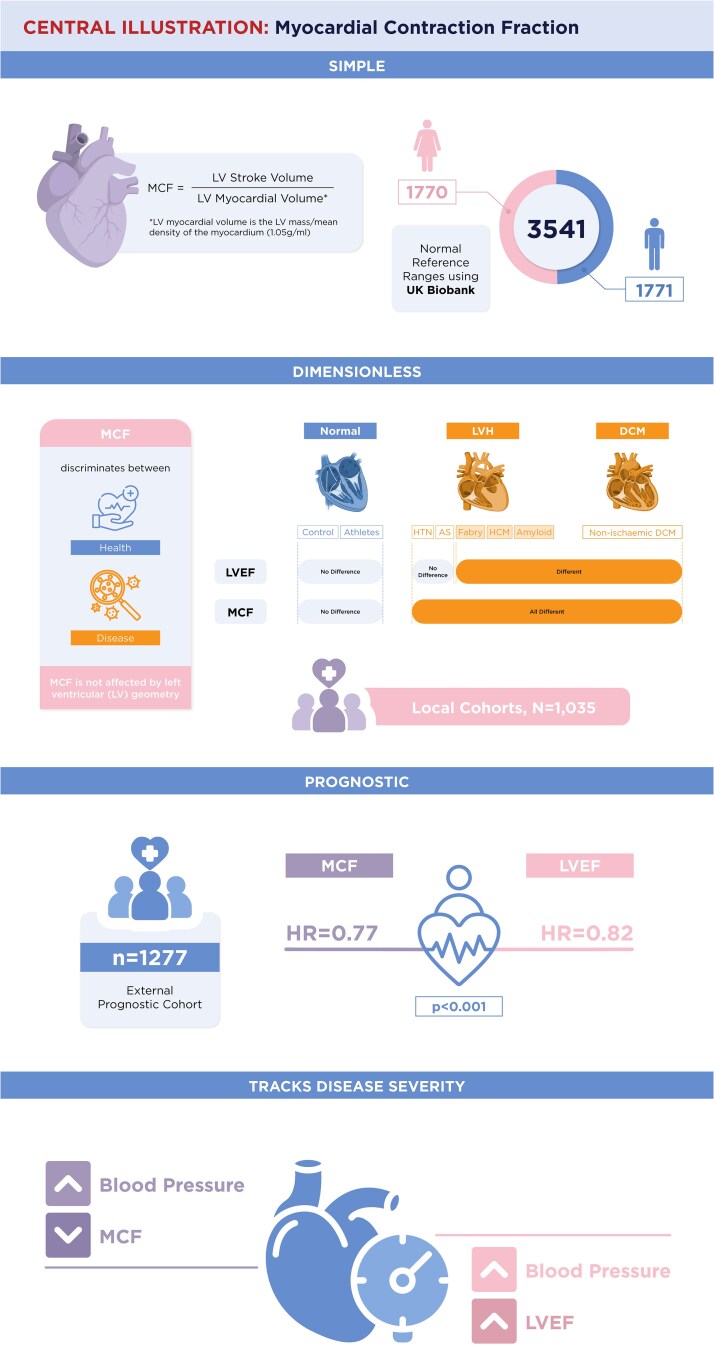
Myocardial contraction fraction (MCF): a simple, dimensionless, and prognostic measure of cardiac function. **Legend**: This central illustration summarizes the key attributes of MCF as a metric for cardiac function assessment. (**Top—Simple**): MCF is derived from stroke volume and myocardial volume, with sex-specific normal reference ranges established from 3541 UK Biobank participants. (**Middle—Dimensionless**): Unlike LVEF, MCF incorporates mass and therefore is susceptible to impact of left ventricular geometry than LVEF and effectively distinguishes between normal, physiological adaptation, and pathological hypertrophy or dilation in 1035 local cohort participants. (**Lower—Prognostic**): In an external cohort of 1277 patients, MCF demonstrates superior prognostic ability compared with LVEF (HR = 0.77 vs. HR = 0.82, *P* < 0.001). (**Bottom—Tracks Disease Severity**): MCF declines with increasing blood pressure, while LVEF paradoxically rises, reinforcing its value as a more sensitive indicator of disease progression.

**See the editorial comment for this article ‘Beyond ejection fraction: back to the myocardium in the assessment of cardiac function’, by E. Donal**  ***et al*****., https://doi.org/10.1093/ehjci/jeag020.**

## Background

Measuring cardiac function is fundamental to cardiovascular disease management, guiding treatment choice,^1^ monitoring disease progression,^2,3^ risk stratification, and prognostication.^4–7^ The left ventricular ejection fraction (LVEF; stroke volume divided by end diastolic volume) is the most frequently used measure of systolic function.^2,3^ LVEF holds prognostic value,^8,9^ with multiple trials demonstrating its ability to predict response to both medical and device therapy.^9,10^ LVEF does, however, have some well-documented limitations.^1,11,12^

The fundamental limitation of LVEF is that it focuses solely on chamber size, overlooking the myocardium, where the pathology typically lies in heart failure and cardiomyopathy conditions. This causes misclassifications—for example, more than half of heart failure patients have preserved ejection fraction (EF).^2,3,12^ In hypertrophic cardiomyopathy^13^ and hypertension (HTN),^14^ LVEF commonly remains within the normal range despite significant myocardial damage and fibrosis. LVEF is often a late marker of disease, such as in cancer therapeutics related cardiac dysfunction.^15,16^ LVEF also overestimates systolic function when the end diastolic volume is small, such as in paradoxical low-flow low-gradient severe aortic stenosis (AS).^2,12,17^

An alternative measure of myocardial performance was proposed over 20 years ago: the myocardial contraction fraction (MCF = ventricular stroke volume divided by myocardial volume).^7^ MCF is simple to compute and is intuitive, representing the stroke volume generated per unit of myocardium. It is dimensionless, avoiding the need for body size correction. It has shown promise in differentiating physiological adaptation from disease^4^ as well as being a better predictor of survival in cardiac amyloid than LVEF.^18,19^ Like LVEF, a lower MCF indicates lower cardiac function.

LVEF can be normal despite a low cardiac output, for example in a small ventricle with concentric hypertrophy.^20^ Whereas, MCF reflects how effectively myocardial tissue generates forward flow.^7,21^

Despite its promise, MCF is underused in clinical practice. One reason could be a lack of a normative reference range, and limited characterization across disease. We address this by establishing a normative reference range for MCF in healthy subjects and investigate its behaviour in different chronic structural cardiac diseases. We then determine the ability of MCF to differentiate health from disease and compare it to the established LVEF. We also investigate how MCF and LVEF tracks worsening disease, using HTN as an exemplar. Finally, we assess its potential as a prognostic biomarker in successive patients referred to a clinical cardiac MR service, comparing it to the established LVEF.

## Methods

### Consent

All subjects were recruited as part of ethically approved research studies and provided written informed consent. All studies were conducted in accordance with the principles of the declaration of Helsinki.

### Data

Unless otherwise stated, all subjects were scanned on Siemens scanners (Aera/Avanto, Siemens Medical Imaging, Erlangen, Germany) at 1.5T using standard steady state free precession sequences.

#### Healthy subjects for deriving a normal reference range

Reference ranges for ‘normal’ values were determined using data from two sources. For both, healthy volunteer (HV) status was determined based on self-reported medical history at the time of recruitment. The first source was 3541 healthy subjects (61 ± 7, 50% male) from the UK Biobank's imaging substudy (project number 71702).^22^ Subjects were considered healthy if they took no medications and were free from HTN, diabetes, hypercholesterolaemia, peripheral vascular disease, coronary artery disease, and cerebrovascular disease. The second group was 121 HVs recruited locally, with a broader age range (45 ± 15 years, 44% male), free of cardiovascular disease and diabetes. A small number took a statin for primary prevention.^23^

**Veteran athletes**: had a mean age of 54 ± 8 years, with 50% male participants. Inclusion criteria required athletes to be over 40 years old, have engaged in competitive endurance exercise for more than 10 years, and have participated in multiple running competitions. Recruitment was conducted across multiple sites in London.^24^

**Hypertension**: Patients aged 18–80 years with primary HTN (57 ± 13 years, 55% male) were recruited from a HTN clinic at a major tertiary referral centre.^25^ Each patient underwent thorough evaluation to exclude secondary HTN as a standard part of their management.

**Fabry disease**: Genetically confirmed Fabry participants (46 ± 15 years, 44% male) over the age of 18 were recruited from a specialist Fabry clinic at the Royal Free Hospital, London.^26^

**Cardiac Amyloidosis**: Patients with wild-type transthyretin cardiac amyloidosis (ATTR) (73 ± 9 years, 90% male) were recruited retrospectively from Barts Heart Centre, London (UK) using the clinical cardiac magnetic resonance (CMR) database ethics (REC 21/EE/0037; IRAS 294495). Diagnosis was based on international guidelines,^27^ and all patients had a Perugini grade of 2–3 on bone scintigraphy (DPD) imaging.

**Hypertrophic cardiomyopathy (HCM)**: Patients with a clinical diagnosis of HCM (52 ± 14 years, 44% male), were recruited from specialist heart muscle disease clinics at The Heart Hospital and Barts Heart Centre, London UK.^28^

**Severe aortic stenosis**: Patients with severe AS (71 ± 10 years, 62% male), with 2 or more of: AVA < 1 cm^2^, peak pressure gradient 64 mmHg, mean pressure gradient 40 mmHg, velocity ratio < 0.25; or reclassification of discordant echocardiographic data to severe by alternate modality were recruited from University College London Hospital.^29^

**Non-ischaemic dilated cardiomyopathy (DCM):** Patients with a diagnosis of non-ischaemic DCM (61 ± 13 years, 66% male) were retrospectively recruited from Barts Heart Centre, London using the clinical CMR database ethics (REC 21/EE/0037; IRAS 294495).

#### Severity of hypertension

The UK Biobank imaging substudy cohort (41 558 subjects; 64 ± 7 years, 48% male) was used to analyse HTN severity, with office blood pressures recorded during the same visit as CMR.^22^HTN was graded according to the 2018 ESC guidelines.^30^

#### Clinical outcome data

For prognostic assessment, we analysed CMR data collected from an independent single-centre multi-disease cohort of consecutive consented patients (University of Pittsburgh Medical Centre, *n* = 1277, 55 ± 15years, 58% male). Clinical outcome data were collected over 5.5 years, with the primary outcome of all-cause mortality occurring in 227 (18%) patients.^31^

### Image analysis

**Figure 1:**
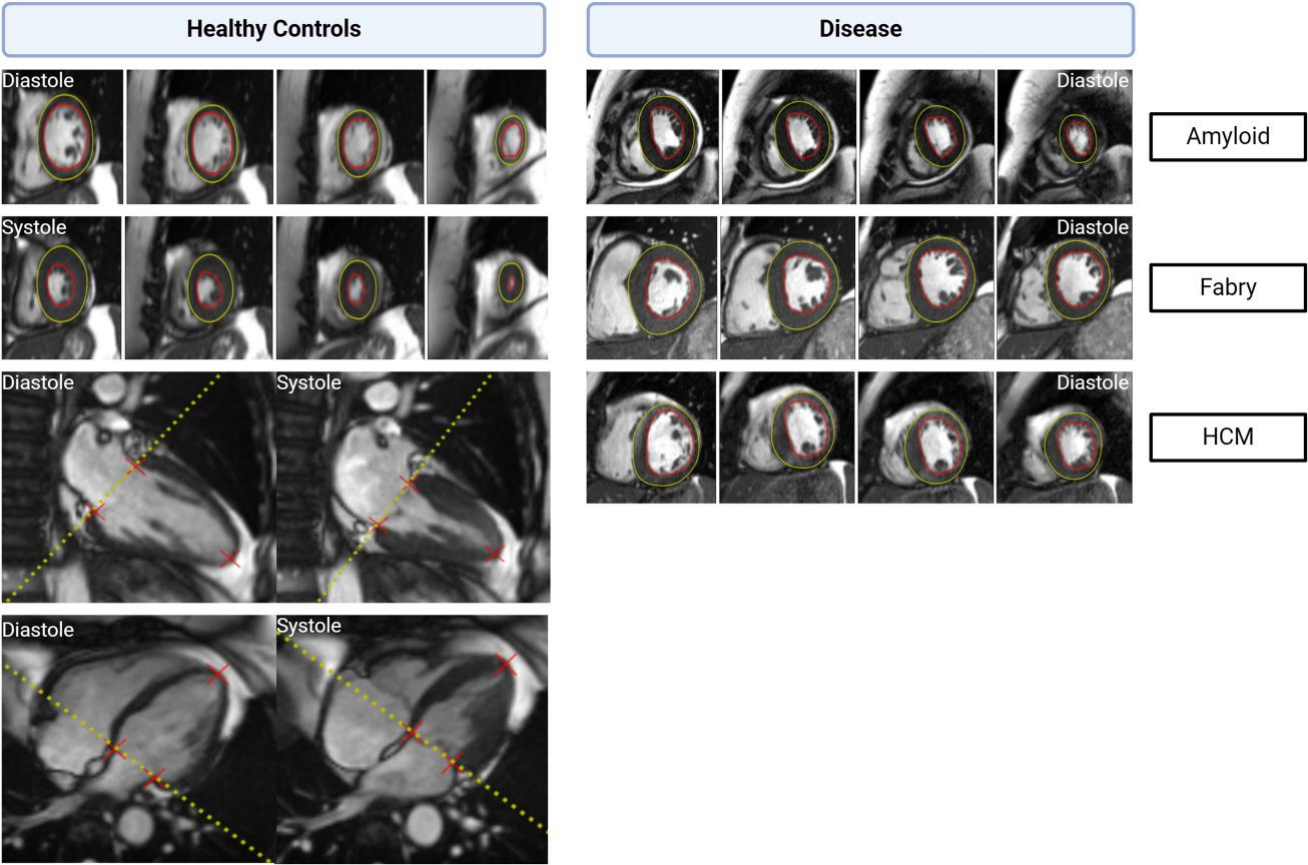
Automated AI-based myocardial segmentation in health and disease. **Legend:** Representative cardiac MRI images showing how the AI tool used in this study segmented the myocardium in healthy controls and example disease states. **Left:** Healthy controls in diastole and systole with normal myocardial contraction. **Right:** Pathological hypertrophy in TTR amyloidosis, Fabry disease, and hypertrophic cardiomyopathy (HCM), showing altered myocardial morphology and contraction patterns in diastole. Green and red contours indicate automated endocardial and epicardial segmentation, respectively. Red crosses mark key anatomical landmarks (mitral valve insertion points and apex) in long axis imaging used for alignment and consistency in myocardial segmentation across diastole and systole.

All CMR images were analysed using a clinically validated artificial intelligence (AI) tool using rounded contours, *Figure 1,* and including the papillary muscles as part of the blood pool.^32^ Left ventricular (LV) volumes were generated from the short axis cine stack, with the apical–basal truncated by a plane fitted to the mitral annulus to ensure anatomically consistent segmentation. The LV outflow tract was incorporated into the segmentation and treated as part of the LV blood volume. All images were verified by a clinician and manual correction was not required in all cases. This facilitated large-scale automated analysis, offering superior precision than manual clinician annotation alone.^32^ Full details of the AI analysis can be found in the paper by Davies et al^32^ and in the Supplementary data online, *see S1*.

### Statistics

Statistical analysis was performed in R using RStudio (v4.3.2). Data normality was assessed visually (histograms, QQ plots) and with the Shapiro–Wilk test. Continuous variables are summarized as mean (±SD) if normally distributed or median [IQR] if skewed and compared using Student’s *t*-test or Mann–Whitney–Wilcoxon test, respectively. Categorical data were presented as counts (%) and compared using the *χ*^2^ test. Correlations were assessed with Pearson’s or Spearman’s coefficients based on linearity and homoscedasticity.

Linear regression was used to evaluate associations between MCF/LVEF, demographics (age, sex), systolic blood pressure (SBP), and cardiac MRI measures. One-way ANOVA with Dunnett’s post-hoc tests compared mean across cohorts against controls. Receiver operating characteristic analysis assessed MCF and LVEF’s ability to distinguish LVH-related diseases from healthy states, with optimal thresholds determined via Youden’s *J* statistic. To assess the influence of loading conditions, nested models incorporating SBP as a surrogate for afterload were applied to LVEF and MCF.

Cox proportional hazards regression (coxph package) evaluated associations between MCF, LVEF, EDVi, LVMi, and SV with all-cause mortality. Hazard ratios (HRs) and 95% confidence intervals (CIs) were calculated, with EF and MCF rescaled to reflect 10% increments (EF_10_, MCF_10_) for clinical relevance. Nested models compared the incremental value of EF and MCF using likelihood ratio tests.

Reference intervals [(mean ± 1.96 × SD)] were established for normal values, and statistical significance was set at *P* < 0.05. MCF was not indexed to body surface area (BSA). Test–retest analysis assessed the reproducibility of machine learning CMR parameters against manual human contouring. Reproducibility was evaluated using Bland–Altman analysis, intraclass correlation coefficients, and mean absolute differences with 95% CIs.

## Results

In total, 46 134 subjects were included and a breakdown of the number of subjects recruited from each cohort along with the age, body size, sex, and LV metrics is reported in *Table 1*.

**Table 1:** Demographic details for each cohort

*Cohort*	UKB (Healthy cohort, n = 3541)	UKB (HTN, n = 41 558)	HV (n = 121)	Veteran Athletes (n = 278)	HTN (n = 40)	Fabry (n = 202)	HCM (n = 164)	Severe AS (n = 76)	DCM (n = 103)	TTR-Amyloid (n = 51)
*Age (years)*	61 (±7)	64 (±7)	45 (±15)	54 (±8)	57 (±13)	46 (±15)	52 (±14)	71 (±10)	61 (±13)	73 (±9)
*Sex(%): male*	50	48	44	50	55	44	63	62	66	90
*BSA (m^2^)*	1.84 (±0.2)	1.63 (±0.6)	1.51 (±0.3)	1.78 (±0.2)	2.0 (±0.2)	1.83 (±0.3)	2.0 (±0.3)	1.92 (±0.2)	2.0 (±0.3)	1.92 (±0.2)
*EDVi (mL/m^2^)*	78 (±13)	76 (±14)	97 (±18)	95 (±17)	72 (±20)	85 (±21)	86 (±46)	83 (±33)	125 (±23)	87 (±19)
*ESVi (mL/m^2^)*	27 (±8)	24 (±8)	32 (±8)	32 (±8)	26 (±13)	26 (±9)	22 (±14)	32 (±27)	83 (±24)	39 (±17)
*SVi (mL/m^2^)*	51 (±8)	53 (±9)	65 (±11)	64 (±12)	46 (±11)	60 (±15)	64 (±34)	50 (±16)	42 (±11)	48 (±13)
*LVMi (g/m^2^)*	53 (±10)	50 (±11)	62 (±13)	61 (±13)	58 (±22)	74 (±37)	94 (±55)	82 (±26)	79 (±18)	104 (±21)
*LVEF (%)*	69 (±6)	69 (±7)	67 (±5)	67 (±5)	65 (±10)	71 (±7)	76 (±8)	64 (±15)	34 (±9)	56 (±13)
*MCF*	1.02 (±0.16)	1.12 (±0.19)	1.12 (±0.18)	1.12 (±0.15)	0.90 (±0.23)	0.97 (±0.28)	0.72 (±0.20)	0.69 (±0.17)	0.58 (±0.20)	0.50 (±0.16)

Values are %, mean ± SD.

AS, aortic stenosis; BSA, body surface area; DCM, dilated cardiomyopathy; EDVi, end diastolic volume indexed; ESVi, end systolic volume indexed; HCM, hypertrophic cardiomyopathy; HTN, hypertension; HV, healthy volunteer; LVEF, left ventricular ejection fraction; LVMi, left ventricular mass indexed; MCF, myocardial contraction fraction; SVi, stroke volume indexed; TTR amyloid, transthyretin amyloid; and UKB, United Kingdom Biobank.

### Normal reference range for MCF

**Figure 2:**
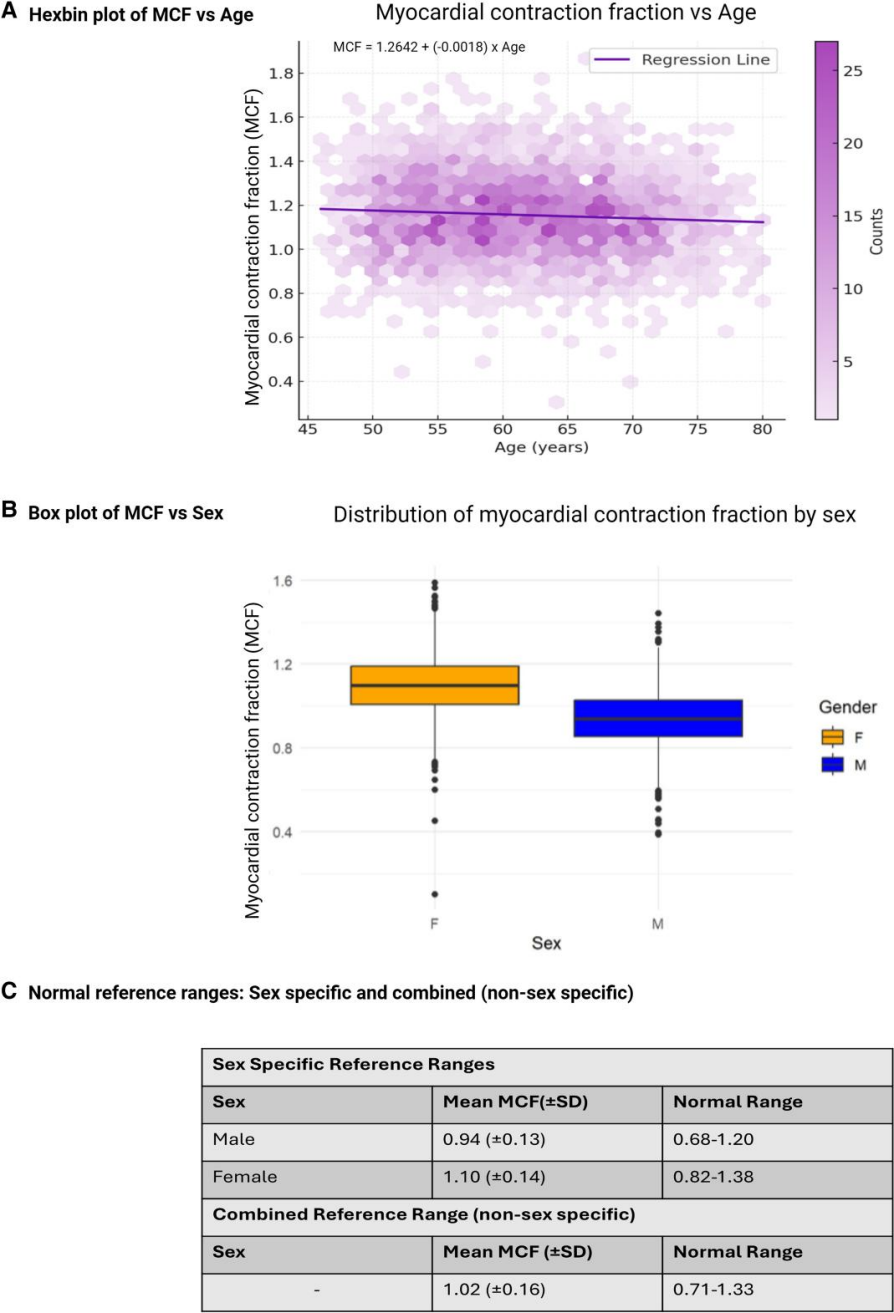
Age and sex variation in myocardial contraction fraction (MCF) and normal reference ranges. **Legend:** (*A*) Hexbin plot showing the relationship between MCF and age, with a fitted regression line indicating minimal association. (*B*) Box plot demonstrating the distribution of MCF by sex, highlighting higher values in females. (*C*) Normal reference ranges for MCF including sex-specific and combined (non-sex specific), derived from the UK Biobank healthy cohort (*n* = 3541).

Reference ranges were derived from 3541 healthy subjects from the UK biobank. There was a weak linear relationship of MCF and age, with MCF decreasing by only 0.002 with every decade, (*r* = −0.0018)—see *Figure 2A*. Minimal model variance (R^2^ = 0.0054) was explained by age for both MCF and EF, Supplementary data online, *S2*, so reference ranges were not conditioned on age.

Females had a significantly higher MCF than males [female 1.10(±0.14) vs. male 0.94(±0.13)]—see *Figure 2B*. Sex-specific reference ranges were therefore derived—see *Figure 2C*. The reference ranges were: [0.68–1.20] for male and [0.82–1.38] for female. To facilitate comparison with external population-based studies, we additionally report a combined (non–sex-specific) reference range for MCF of 0.71–1.33, *Figure 2C*.

When indexed to BSA, this MCF sex difference persists [female 0.73(±0.12) vs. male 0.55(±0.09)]. Box plots of indexed MCF stratified by sex are included in the Supplementary data online, see *S3*.

### MCF behaviour in physiological adaptation and across disease

A summary of the demographics of each cohort is tabulated in *Table 1*.

There was no significant difference between health and physiological adaptation for MCF or LVEF: mean MCF: health (HV) 1.12, athletes 1.12; LVEF: health (HV): 67%, athletes 67%.

**Figure 3:**
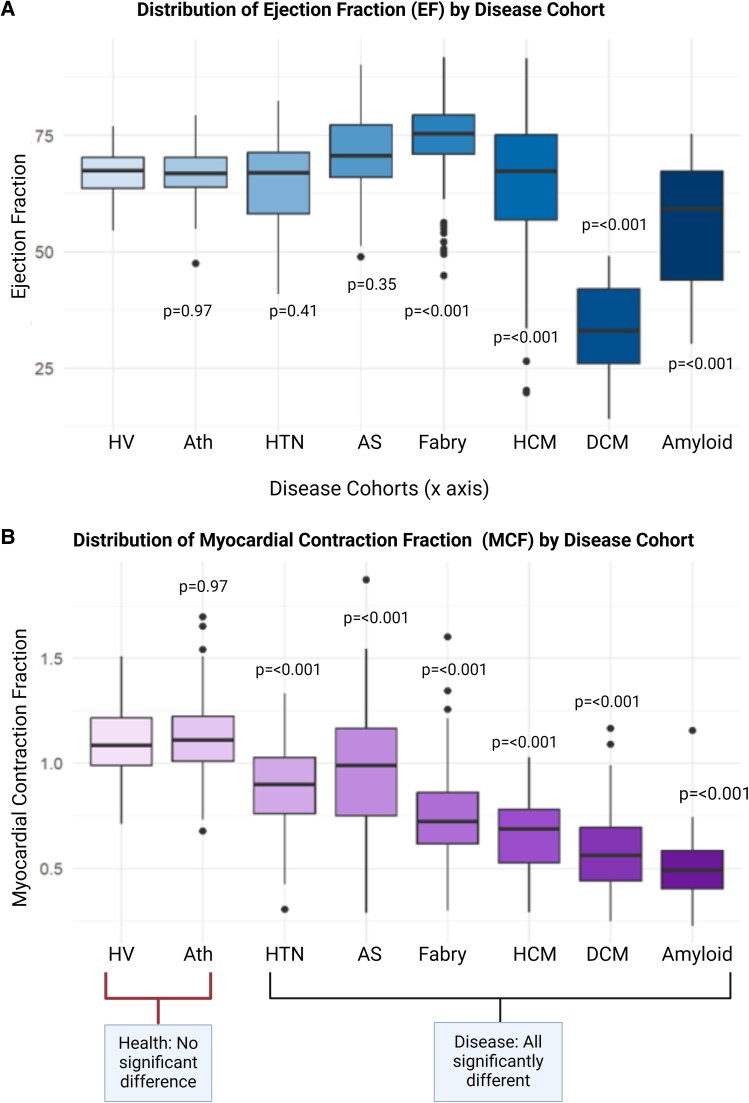
Comparison of ejection fraction (EF) and myocardial contraction fraction (MCF) across health and disease. **Legend:** (*A*) Box plot showing the distribution of EF across different cohorts (*x* axis), demonstrating significant reductions only in amyloidosis, hypertrophic cardiomyopathy (HCM), and dilated cardiomyopathy (DCM). (*B*) Box plot of MCF across the same cohorts, illustrating significantly lower values in all disease states compared with healthy volunteers (HVs) and veteran athletes (Ath). The lower panel highlights that MCF distinguishes disease states more effectively than EF.

MCF was significantly reduced across all disease states (HTN 0.90 ± 0.23, Fabry 0.97 ± 0.28, HCM 0.72 ± 0.20, AS 0.69 ± 0.17, DCM 0.58 ± 0.20, ATTR amyloid 0.50 ± 0.16) and was significantly different across all disease states (all *P* < 0.05). LVEF showed limited discrimination between health and disease, with no significant reduction in all disease cohorts except ATTR-amyloid and DCM (%: HTN 65 ± 10, Fabry 71 ± 0.28, HCM 76 ± 10, AS 64 ± 15, DCM 34 ± 9, ATTR amyloid 56 ± 13), *Figure 3*.

LVEF and MCF demonstrated a modest positive correlation (*r* = 0.36, *P* < 0.001), with LVEF explaining 12.6% of the variance in MCF (*R*^2^ = 0.126), see Supplementary data online, *S4*.

### Blood pressure and loading conditions

SBP was used as a surrogate for afterload in the UK Biobank cohort. Lack of complete SBP results for all cohorts limits further assessment. Univariable linear regression models showed a marginally higher *R*^2^ for the association between MCF and SBP (*R*^2^ = 0.039, *P* < 0.001) compared with LVEF and SBP (*R*^2^ = 0.018, *P* < 0.001), Supplementary data online, *S5A*. However, in multivariable models adjusted for age and sex, the association between MCF and SBP retained a weak negative characteristic (standardized *β* = −0.089) while LVEF retained a positive association (*β* = 0.161). In nested models, adding SBP reduced the residual sum of squares (ΔSS) by 303 units in the LVEF model (*P* = <0.05), compared with 0.15 units in the MCF model (*P* = <0.05). *See*  Supplementary data online, *S5B*.

#### Tracking disease severity

**Figure 4:**
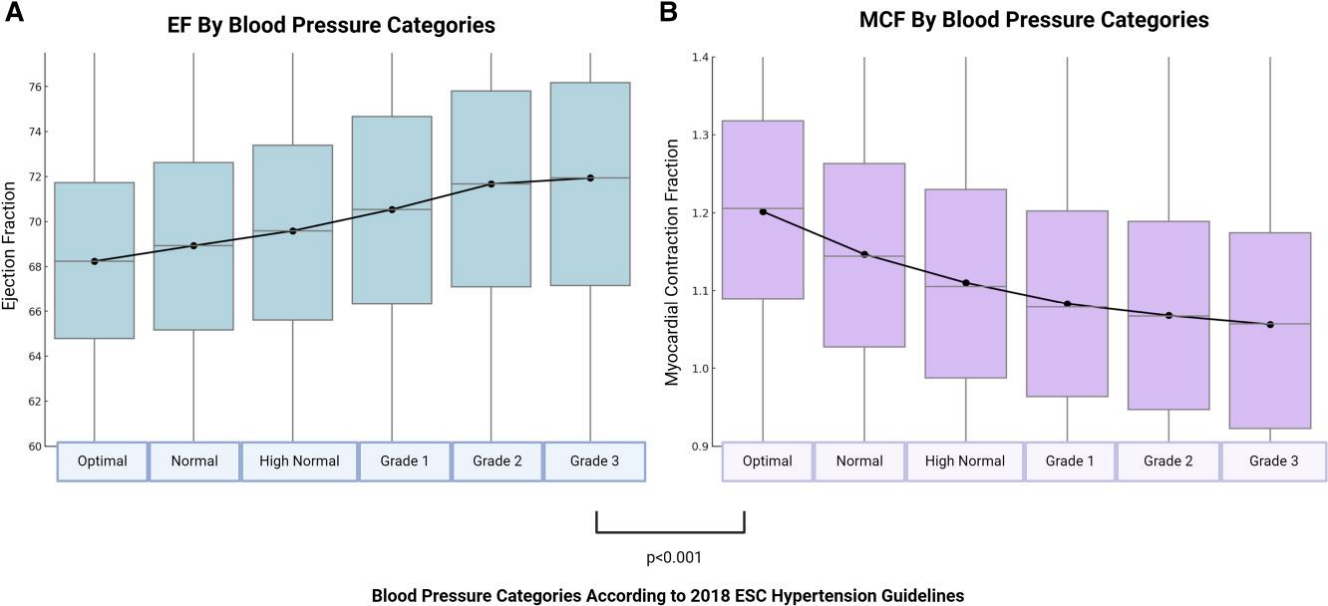
Diverging trends of ejection fraction (EF) and myocardial contraction fraction (MCF) across blood pressure categories. **Legend:** Box plots (mean ± SD, black dots representing mean) demonstrating trends in EF (*A*) and MCF (*B*) with blood pressure categories. EF increases with rising blood pressure and MCF declines progressively, suggesting it better reflects myocardial dysfunction. Trends are significant (*P* < 0.001).

*Figure 4A* plots the relationship between grades of HTN and LVEF, paradoxically showing a significant (*P* < 0.001) increase in LVEF as HTN becomes more severe. MCF shows a significant (*P* < 0.001) decrease in myocardial performance with increasing grades of HTN *Figure 4B*.

#### MCF for disease discrimination and prognostication

**Figure 5:**
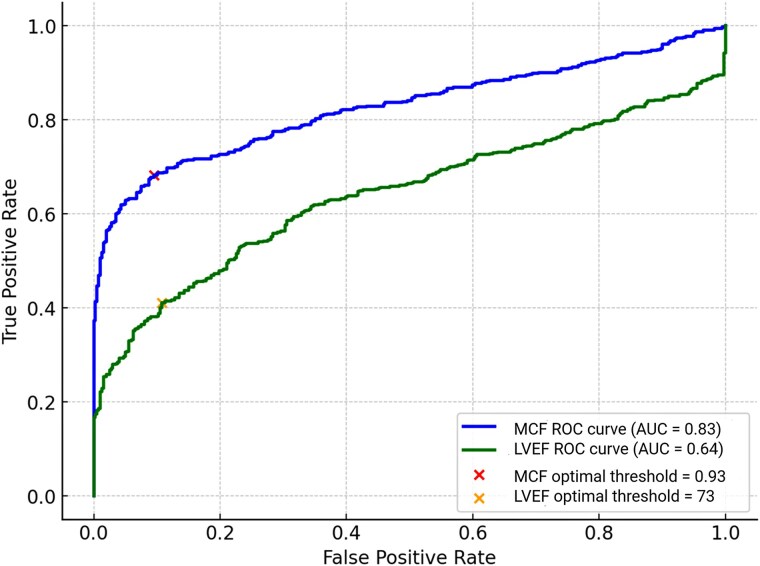
Receiver operating characteristic (ROC) curve showing the differentiation between health (healthy volunteers and veteran athletes) and LVH diseases using myocardial contraction fraction (MCF) and left ventricular ejection fraction (LVEF). **Legend:** MCF (blue) demonstrates superior performance (AUC = 0.83, 95% CI: 0.800–0.853, *P* < 0.001) compared with LVEF (green, AUC = 0.64, 95% CI: 0.607–0.677, *P* < 0.001). The optimal classification thresholds calculated using Youden’s *J* statistic are marked (MCF = 0.93, LVEF = 73), indicating the point at which disease is more likely to be defined, e.g. patient are more likely to have disease with an LVEF of ≤73%.

MCF is better able to discriminate between health and disease, (MCF AUC: 0.83, 95% CI: 0.800–0.853 vs. LVEF AUC: 0.64, 95% CI: 0.607–0.677), *Figure 5*.

The prognostic ability of LV metrics is summarized in *Table 2*. MCF_10_ demonstrated a stronger association with all-cause mortality (HR 0.772; *χ*^2^ = 198) than LVEF_10_ (HR 0.816; *χ*^2^ = 151) and all other LV metrics (*P* < 0.001; *Table 2a*). In multivariable models adjusted for age and sex, MCF remained a robust predictor of mortality (HR 0.33, *χ*^2^ = 70, *P* < 0.001), outperforming LVEF (HR 0.63, *χ*^2^ = 26, *P* < 0.001). Adding MCF to nested models with LVEF, age, and sex significantly improved fit (*χ*^2^ = 53.90, *P* < 0.0001), whereas adding LVEF to MCF-based models provided minimal improvement (*χ*^2^ = 7.10, *P* = 0.07; *Table 2d*).

**Table 2:** Univariable and multivariable analyses of cardiac parameters for all-cause mortality composite endpoint, including nested models with likelihood ratio chi-squared tests

Variable	Hazard ratio	95% CI	*P* value	Chi squared
**(A) Univariable analysis**				
*LVEF_10_	0.82	0.755–0.88	<0.001	151
*MCF_10_	0.77	0.727–0.82	<0.001	198
EDVi	1.01	1.003–1.01	<0.001	11
LVMi	1.02	1.017–1.03	<0.001	70
SV	0.99	0.981–0.99	<0.001	28
**(B) Multivariable model for EF adjusted for age and sex**				
LVEF	0.63	0.43–0.91	<0.0001	26
Age	2.26	1.49–3.43	<0.0001	95
Sex	1.41	1.06–1.89	0.0179	6
**(C) Multivariable model for MCF adjusted for age and sex**				
MCF	0.33	0.21–0.50	<0.001	70
Age	2.15	1.42–3.26	<0.001	69
Sex	1.18	0.88–1.57	0.2743	1
**(D) Nested models using likelihood ratio chi squared test**				
Comparison	Base model 1	Expanded Model	*P*-value	Chi Squared
	Age, sex, MCF	Age, sex, MCF, LVEF	0.07	7.10
Comparison	Base model 2	Expanded Model	*P*-value	Chi Squared
	Age, sex, LVEF	Age, sex, LVEF, MCF	<0.0001	53.90

*MCF and LVEF included in the Cox proportional hazards models as continuous variables. To enhance clinical relevance and interpretability, both variables were rescaled to represent a 10% change, referred to as LVEF_10_ and MCF_10_, respectively. This transformation allows the hazard ratios to reflect the change in risk associated with a 10% absolute change in LVEF or MCF, rather than a 1% change, which might be less meaningful in clinical practice.

LVEF, left ventricular ejection fraction; MCF, myocardial contraction fraction.

#### Reproducibility of MCF assessment using machine learning and human contouring

Test–retest precision comparing automated (machine learning) and manual (human) cardiac MRI measurements are reported in Supplementary data online, *Table S6*.

## Discussion

The LVEF remains the cornerstone of clinical decision making in cardiovascular medicine, despite long-recognized limitations in its ability to reflect true myocardial contractile performance.^1^ In particular, LVEF may remain normal even in the presence of substantial disease burden in conditions characterized by concentric remodelling or infiltration, where cavity size is reduced relative to myocardial mass. In this study, we evaluated the MCF, a volumetric ratio of stroke volume to myocardial volume and demonstrate that it provides a more physiologically grounded and clinically informative measure of global systolic function across diverse disease states.

A major limitation of LVEF is its dependence on ventricular geometry, because it expresses stroke volume relative to end-diastolic volume. In concentric remodelling, such as hypertensive heart disease^33^ or AS,^6,33^ the disproportionate reduction in cavity size can yield deceptively normal or even elevated LVEF despite impaired myocardial shortening.^34–36^ In contrast, MCF relates stroke output to the volume of contracting myocardium, offering a geometry-independent assessment of myocardial pump efficiency.^7,21^ Both stroke volume and myocardial volume scale proportionally with body size,^37–40^ meaning their ratio is dimensionless and inherently normalized. We showed that the sex differences persist despite indexing to body size in keeping with previous work that showed that both stroke volume and myocardial volume scale proportionally with body size.^37–40^ We therefore elected not to index MCF to BSA in order to preserve its simplicity and inherently dimensionless nature, consistent with prior studies and with the convention used for LVEF.

Our findings extend earlier work showing that MCF identifies subclinical dysfunction and predicts clinical outcomes in both population studies^41^ and in disease-specific settings such as cardiac amyloidosis,^18,19^ AS,^42–44^ and hypertrophic cardiomyopathy.^5,45^ Here, using large and generalizable datasets from the UK Biobank and a US prognostic cohort, we confirm that MCF offers broader discriminatory power than LVEF, and we establish sex-specific reference ranges using automated CMR analysis validated across independent populations. Because MCF is derived from routinely reported CMR metrics, it requires no additional imaging sequences, geometric assumptions, or manual contouring, making its clinical implementation straightforward.

Across the external prognostic cohort, MCF and LVEF were both associated with all-cause mortality in univariable analyses; however, MCF provided the strongest prognostic discrimination (*χ*^2^ = 198), exceeding that of LVEF (*χ*^2^ = 151), LV mass index (*χ*^2^ = 70), and LVEDVi (*χ*^2^ = 11). Notably, stroke volume and myocardial mass alone did not consistently predict outcomes, highlighting the physiological significance of their ratio rather than their absolute values. These results indicate that MCF captures integrated, disease relevant properties beyond its components, supporting its role as a more comprehensive marker of contractile efficiency.

Global longitudinal strain (GLS) is widely used as a deformation based index of myocardial performance and can detect early systolic impairment across a broad range of diseases, including HFpEF,^46^ AS,^47^ Fabry disease (FD),^48^ and cancer therapy related cardiotoxicity.^49^ Several studies have reported moderate to strong correlations between MCF and GLS.^44^ In hypertrophic cardiomyopathy with preserved LVEF, both GLS and MCF independently predict myocardial fibrosis on late gadolinium enhancement,^50^ supporting their ability to detect subclinical disease despite normal EF. Although few studies have directly correlated the two indices;^44^ the available evidence shows that MCF performs at least as well as GLS and in some settings, such as low-gradient AS,^6^ may even offer superior prognostic value.^51^

Emerging echocardiographic pressure-strain loop derived myocardial work indices add further nuance. In a large population study, both GLS and LVEF declined with increasing HTN severity, whereas myocardial work indices increased, in keeping with the MCF results reported here.^51^ This divergence highlights the afterload sensitivity of both deformation and volume-based measures and suggests that myocardial work may be less load dependent. Two-thirds of participants with abnormal GLS had normal myocardial work indices,^51^ illustrating substantial discordance between commonly used functional measures. Collectively, these findings reinforce the need for complementary indices to capture the multidimensional behaviour of myocardial systolic function.

Against this backdrop, MCF offers conceptual advantages. GLS reflects primarily longitudinal fibre deformation and is therefore also sensitive to loading conditions, particularly afterload. In contrast, MCF integrates stroke performance relative to total contracting myocardium, a global measure less influenced by ventricular shape or geometry. MCF may therefore reveal physiological impairment in cases where GLS is preserved or where loading conditions obscure deformation indices.^52^

LVEF depends on chamber size and shape, making it sensitive to ventricular geometry, and prone to misrepresenting myocardial contractility.^52^ LVEF, for example, can overestimate contractility in small ventricles with concentric hypertrophy.^20^ Strain, which measures tissue deformation, is less influenced by geometry.^52^ Since the denominator for MCF is myocardial volume rather than end diastolic volume, it too is less dependent on cardiac geometry and more reflective of myocardial contraction. Further direct comparisons across disease states and haemodynamic conditions will be essential to delineate the unique and overlapping roles of MCF, GLS, and myocardial work within contemporary imaging practice.

To explore load sensitivity directly, we examined the relationship between SBP and both LVEF and MCF within the UK Biobank subset. LVEF increased with rising SBP, consistent with compensatory mechanisms that preserve stroke output under elevated afterload. Conversely, MCF showed a mild but directionally appropriate decline with increasing SBP, indicating a more physiologically coherent response to rising haemodynamic load. Although modest, these findings support the concept that MCF may better reflect true contractile performance rather than haemodynamic compensation. Nevertheless, because these observations are limited to a subset of middle-aged and older adults, they warrant validation in broader cohorts.

Several approaches could help refine the understanding of MCF under varying physiological conditions. Concurrent measurement of central blood pressure, pulse-wave velocity, and aortic distensibility could allow indirect modelling of afterload^53^ relative to MCF. Combining MCF with myocardial strain may disentangle the load-dependent and load-independent components of systolic performance, particularly given prior studies demonstrating strong correlations between these measures.^53^ Additionally, serial imaging under controlled interventions, such as volume loading, vasodilation, or alteration of posture could clarify the dynamic behaviour of MCF under acute haemodynamic shifts. These strategies will be important to define how MCF should be interpreted across the spectrum of patient presentations.

Disease-specific factors also require consideration. Because MCF, like LVEF, is derived from total stroke volume, significant mitral or aortic regurgitation may lead to overestimation of effective forward systolic performance. This limitation mirrors LVEF behaviour in regurgitant lesions, where clinical guidelines use disease-specific EF thresholds rather than mathematically correcting the measure for regurgitant volume. Although deriving MCF from aortic forward flow could theoretically address this, it would constitute a distinct metric requiring validation and standardization. Until such studies are undertaken, MCF should be interpreted cautiously in contexts of significant regurgitation, especially when forward flow is clinically relevant.

The implications of our findings extend to clinical pathways that rely heavily on LVEF. Many therapeutic decisions including initiation of guideline directed medical therapy, selection for device therapy, and referral for advanced heart failure management, depend on LVEF cut-off points. However, LVEF frequently fails to identify impaired contractility in conditions grouped under the HFpEF umbrella,^12^ particularly hypertensive heart disease and amyloidosis. Incorporating MCF may reclassify patients currently labelled as having preserved systolic function and enable earlier risk stratification or intervention.^54^ Further work is needed to define the diagnostic and prognostic value of MCF in this population. To achieve this, additional data are required, including confirmed HFpEF diagnoses, natriuretic peptide levels and clinical outcomes. We emphasize that MCF is not yet positioned as a replacement for LVEF but as a complementary metric that can enhance clinical decision making, particularly in cases where LVEF is preserved but physiological suspicion remains high.

Our findings differ from earlier echocardiographic observations such as the Framingham Heart Study,^55^ which reported modest age-related declines in MCF. In contrast, our CMR-based analysis found MCF to be relatively stable with ageing beyond early adulthood. This discrepancy may reflect differences in imaging modality, measurement reproducibility, or population characteristics. Sex differences were consistent, with women exhibiting higher MCF than men, in keeping with previous reports^55^ and the known tendency for women to have smaller LV cavities even after indexing for body size.^56^ These findings may be clinically meaningful given the higher prevalence of HFpEF among women^57^ and prior data suggesting that MCF is more effective for detecting systolic impairment in HFpEF than in HFrEF.^4^

A major strength of this study is the use of AI-enabled CMR segmentation, which supports scalability and clinical translation.^32^ However, reliance on CMR limits immediate applicability to settings where echocardiography is the primary modality. While three-dimensional echocardiography can estimate MCF without geometric assumptions, its precision is lower due to operator dependence and image quality variability.^58^ As AI-based automated quantification becomes increasingly mainstream in echocardiography, reproducibility is expected to improve, enabling cross-platform harmonization of MCF measurement. Ultimately, validation across multiple imaging modalities will be required for widespread implementation.

To facilitate integration into imaging workflows, MCF could be automatically calculated and displayed within routine post-processing software for both CMR and echocardiography. Because both stroke volume and LV mass are already derived from standard analyses, incorporation of MCF requires no additional acquisition time and minimal software modification. To support this transition, we present normal reference ranges and disease-specific distributions, complementing earlier work by Arenja et al.^4^

In order to gain further clinical utility, it would be valuable to establish more granular thresholds (mild/moderate/severe) to contextualize a given MCF. The simplest way to establish these thresholds would be to use statistical methods such as quantiles in healthy populations.^59^ However, this should also be clinically meaningful and a predictor of poor clinical outcome or a positive response to treatment, similarly to that already established for LVEF. We did not feel that the data was strong enough to warrant proposing such granular thresholds, so we do not propose any here. Furthermore, it would be useful to establish a ‘delta’ value that represented a *significant* change in MCF between two scans. Two things should be considered when establishing this value: the minimal detectable change in MCF between two scans and be related to a clinical change. Neither data were available to us so we have not proposed such a value here.

The MCF is therefore not a panacea. Like all volumetric indices, it remains sensitive to preload and afterload to variable degrees,^53^ and may fluctuate with fluid status, posture, or physiological stress.^2,60^ Moreover, MCF is an indirect estimate of systolic performance based on volumetric surrogates, whereas strain imaging offers a more direct measure of myocardial shortening.^52^ Despite these limitations, LVEF itself is load dependent and yet remains integral to clinical practice due to decades of evidence and integration into treatment pathways.^10^ Within this context, MCF should be viewed as a valuable emerging tool that can augment, not replace LVEF and strengthen current strategies for diagnosing, risk stratifying, and managing myocardial disease.

## Limitations

The diseases investigated in this study were not uniformly defined across all stages, including conditions like FD that is heterogenous in nature and affected not only by mutation but also by treatments including enzyme replacement therapy.^61^ We followed convention and used rounded contours to delineate the endocardial border, but MCF values will be different if trabecular segmentation is used.^62^

No comparison was made to myocardial strain or other measures such as myocardial work, and we did not explore load-dependent co-factors such as left atrial volumes. Further work is needed to compare MCF to these other measures as well as further analysis of load-dependency of these different metrics.

We did not propose granular thresholds to contextualize the severity of disease, nor did we establish the difference in MCF between two scans that would represent a significant change. The cut-offs for LVEF, for example, have evolved over time and the severe threshold corresponds to trial evidence of drug and device efficacy, but the other cut-offs are not without controversy.^63^

Like LVEF, MCF should be interpreted with caution in the presence of regurgitant valvular lesions since these will affect the stroke volumes calculated from contouring the LV blood pool. Alternative methods, such as measuring net forward stroke volume from flow imaging (e.g. using phase contrast in CMR) could be used instead, but this was not explicitly studied here, and further work is required to understand the behaviour and utility of MCF in aortic and mitral regurgitation.

The populations used for this study comprised individuals with chronic and stable cardiovascular disease; therefore, the behaviour and sensitivity of MCF in acute conditions such as myocardial infarction or myocarditis, where myocardial composition and loading conditions evolve rapidly, remain uncertain and require dedicated evaluation.

Finally, although we found no significant change in MCF with age, the UKB cohort on which this was investigated only included patients aged 45 or older, so we cannot be certain that this trend extends to younger subjects.

## Conclusion

In conclusion, MCF is a reproducible, scalable, and physiologically grounded measure of myocardial performance. We define sex-specific reference ranges and show that MCF outperforms LVEF in distinguishing health from disease, tracking pathological progression, and predicting adverse outcomes. MCF is intuitive and easy to derive without additional image analysis, so should be reported routinely to complement the LVEF.

Supported by validated automation and consistent prognostic associations, MCF represents a valuable adjunct to conventional systolic assessment. Additional research is required to refine disease-specific MCF thresholds, define the magnitude of change that is clinically meaningful, assess its utility in acute cardiac conditions, HFpEF, and regurgitant valvular disease, and determine the incremental value of MCF beyond established measures such as GLS.

## Supplementary Material

jeag019_Supplementary_Data

## Data Availability

The data that support the findings of this study are available from the corresponding author upon reasonable request.
