# Supplementary material for: Reappraising cardiac function with myocardial contraction fraction: normal values, disease detection, and prognostication
Source: Eur Heart J Cardiovasc Imaging. 2026 Jan 22;27(4):803–16. doi: 10.1093/ehjci/jeag019 (PMC13021279; doi:10.1093/ehjci/jeag019)
Supplement: jeag019_Supplementary_Data [file jeag019_supplementary_data.zip › Supplement.docx]

S1. A description of artificial analysis methods used for contouring and segmentation of the left ventricle.

“There are two approaches that can be used for delineating the blood pool and heart myocardium: either using a rounded endocardial contour to approximate the endocardial border and including the papillary muscle as part of the blood pool, or a trabecular segmentation where papillary muscles and trabeculae are separately delineated and included as part of the myocardial volume.(1) In this work, we estimated rounded contours of the endocardial and epicardial borders using an AI algorithm.(2) The resulting volumes are ‘sliced’ by the mitral annular plane, which is estimated from two intersection points of the mitral valve plane and the myocardium in both the 2-chamber and 4-chamber views. The left ventricular outflow tract is included in the short-axis segmentation but can be truncated by the mitral annular plane.

For full details, including the architecture, image pre-processing and training algorithm the reader is referred to the paper by Davies et al,(2) but in brief 2d U-net models that segment the endocardial and epicardial borders of the LV for all cardiac phases in a short axis stack. Another 2d U-net is used to identify the mitral valve plane points using a heatmap regression approach. The model was trained with the goal of being generalisable across different patients and scanners by including images from multiple centres (13 institutions; 10 scanner models) with multiple pathologies. The model has been shown to improve on clinician test-retest precision.(2,3) “

References

1. Moody WE, Hudsmith LE, Holloway B, Treibel TA, Davies R, Kozor R, et al. Variation in cardiovascular magnetic resonance myocardial contouring: Insights from an international survey. Vol. 50, Journal of Magnetic Resonance Imaging. John Wiley and Sons Inc.; 2019. p. 1336–8.

2. Davies RH, Augusto JB, Bhuva A, Xue H, Treibel TA, Ye Y, et al. Precision measurement of cardiac structure and function in cardiovascular magnetic resonance using machine learning. J Cardiovasc Magn Reson. 2022 Mar 10;24(1):16.

3. Augusto JB, Davies RH, Bhuva AN, Knott KD, Seraphim A, Alfarih M, et al. Diagnosis and risk stratification in hypertrophic cardiomyopathy using machine learning wall thickness measurement: a comparison with human test-retest performance. Lancet Digit Health. 2021 Jan;3(1):e20–8.

S2. Linear regression plot illustrating the relationship between age and left ventricular ejection fraction (EF)


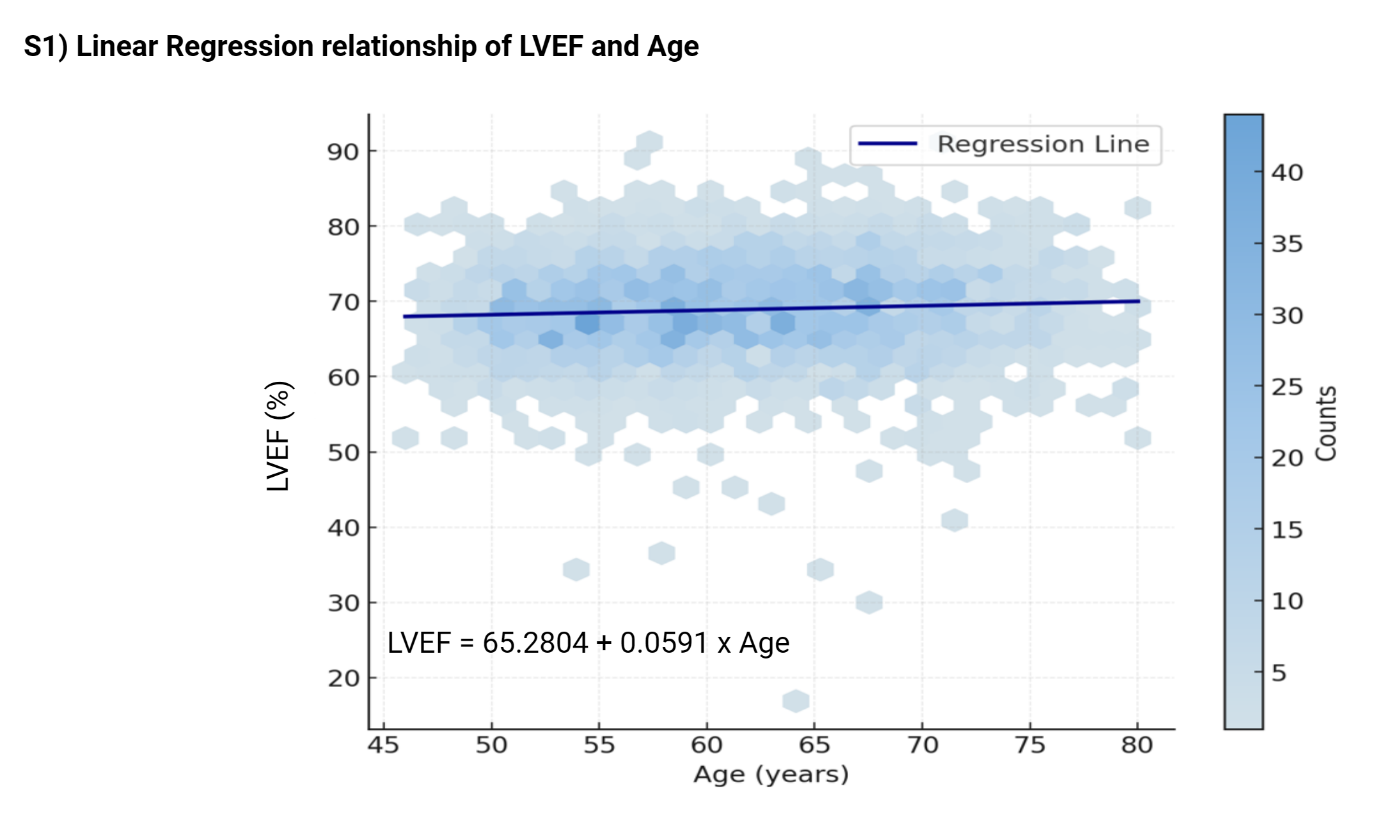


Caption: Using UK Biobank cohort (n= 3,541). Linear Regression Analysis of Ejection Fraction (LVEF) and Age: A weak positive association observed (EF = 65.2804 + 0.0591 × Age), p<0.001. The hexbin plot represents EF distribution across different ages, with darker regions indicating higher data density.

S3. Sex differences in indexed myocardial contraction fraction


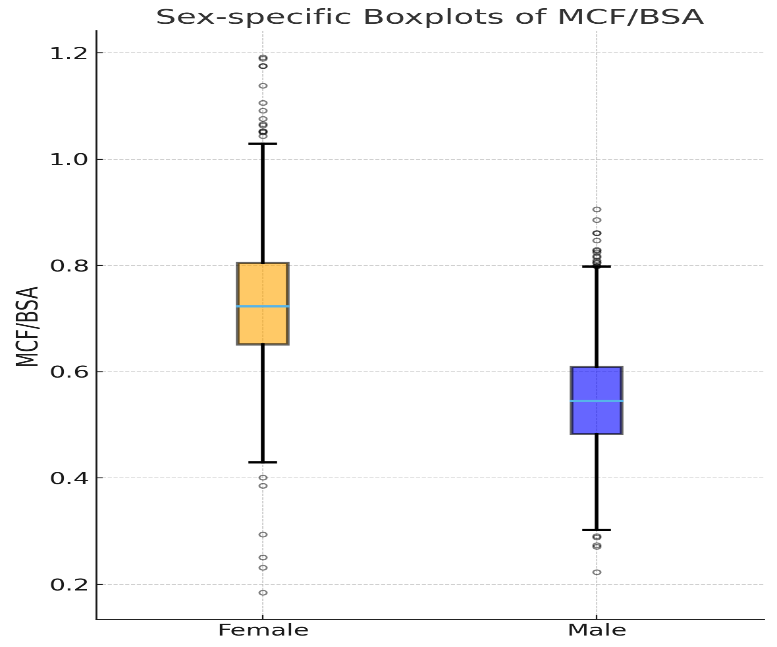


Caption: Sex-stratified boxplots of indexed myocardial contraction fraction (MCF/BSA), showing higher median values and wider distribution in females compared with males. Females: mean MCF of 0.73(±0.12), males 0.55(±0.09). Indexing to BSA did not eliminate the sex distinction.

S4. Linear regression plot illustrating the relationship between LVEF and MCF


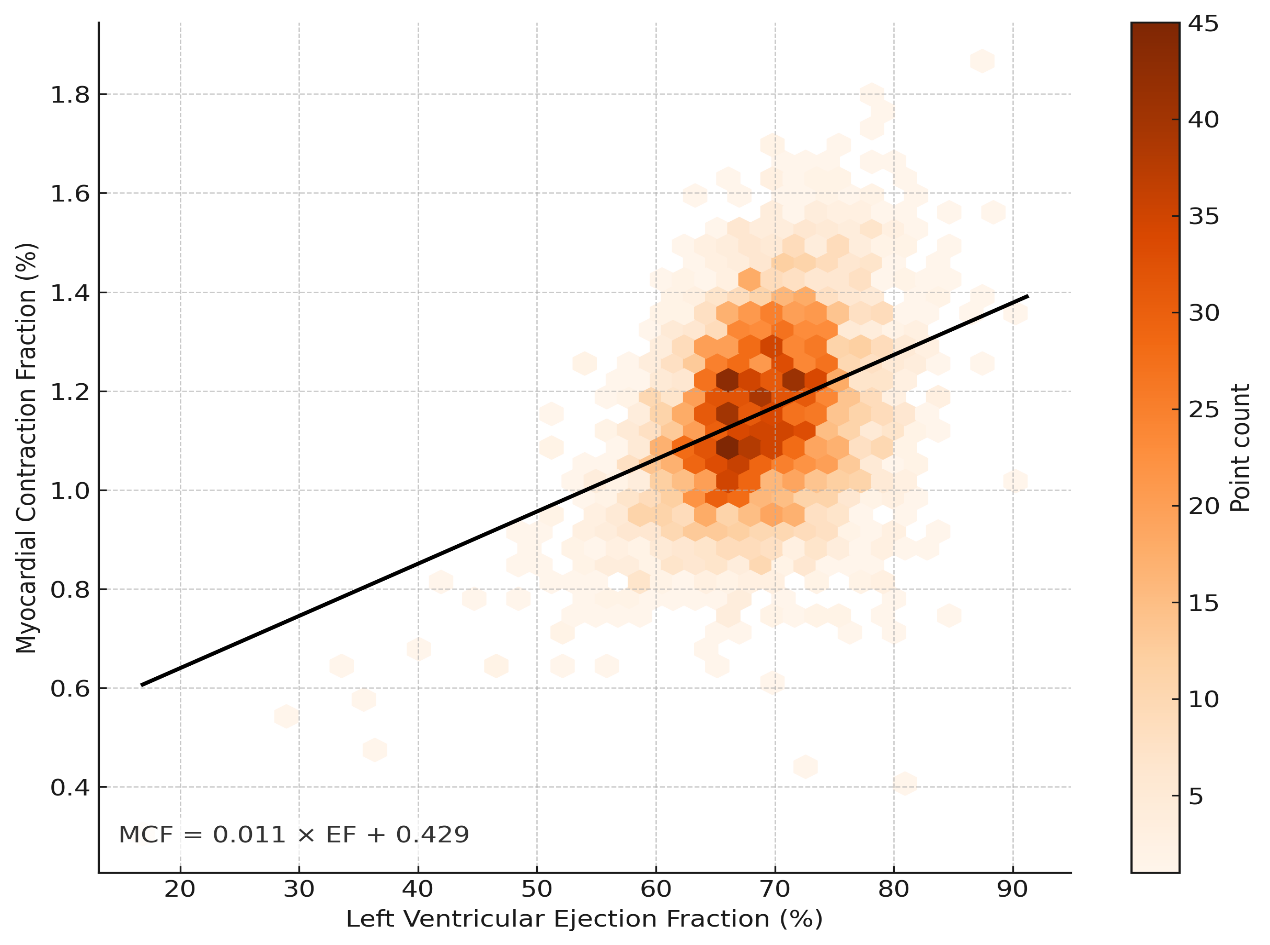


Caption: Using UK Biobank cohort (n= 3,541). Linear Regression Analysis of Ejection Fraction (LVEF) and Myocardial Contraction Fraction (MCF). LVEF and MCF demonstrated a modest positive correlation (r = 0.36, p < 0.001), with LVEF explaining 12.6% of the variance in MCF (R² = 0.126). The hexbin plot represents the distribution of MCF relative to LVEF, with darker regions indicating higher data density.

S5. Linear regression plots illustrating the relationship between systolic blood pressure and LVEF and MCF (A) and table of linear regression and nested models (B)

**A)**


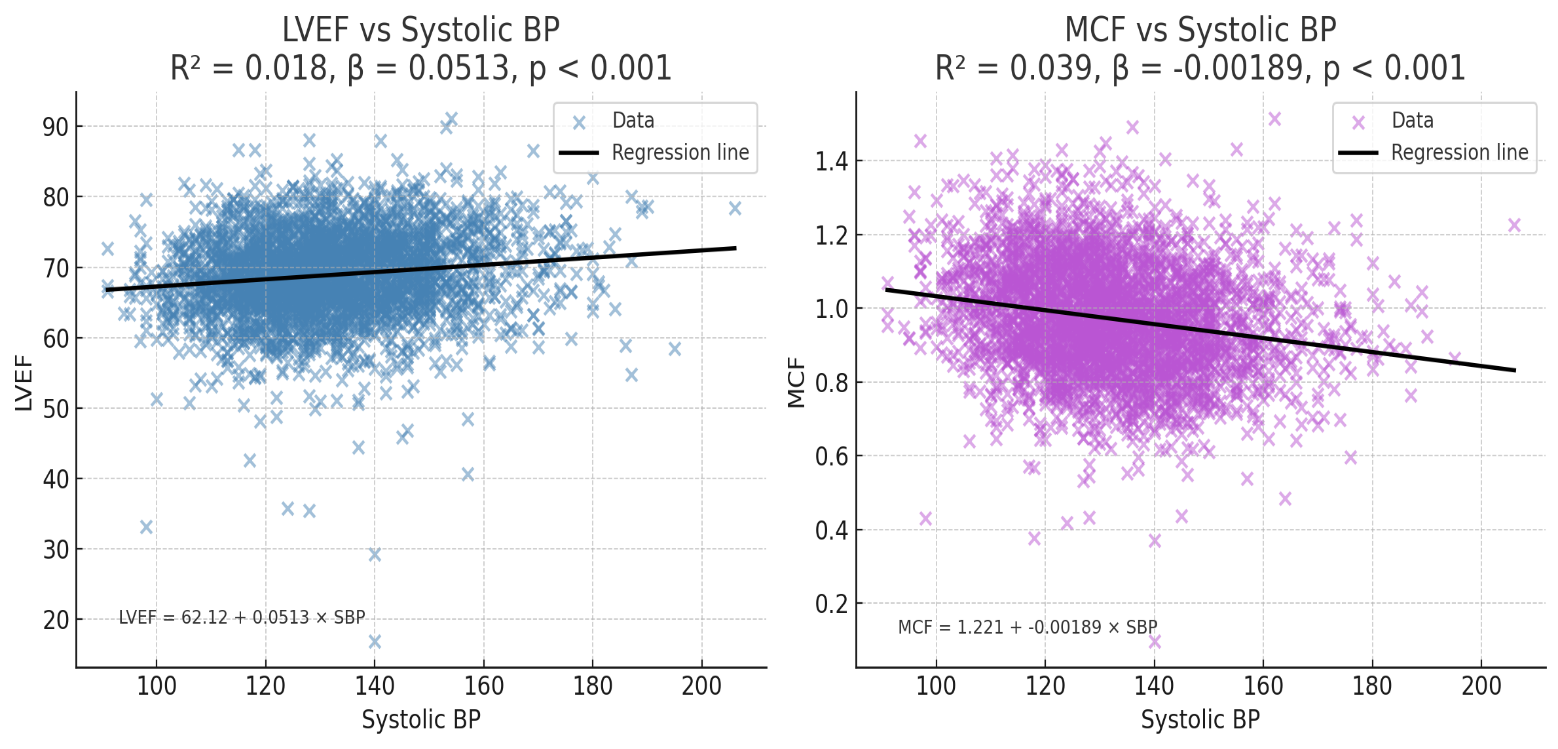


**B)**

| Multivariable linear model adjusted for age and sex | | | |
| --- | --- | --- | --- |
| **Variable** | **Standardised β (SBP)** | **R^2^** | **p value** |
| LVEF | 0.161 | 0.0476 | <0.001 |
| MCF | -0.089 | 0.257 | <0.001 |
| Nested models | | | |
| Base model 1 | Expanded model 2 | ΔSS | p value |
| LVEF, age, sex | LVEF, age, sex, **SBP** | 303 | 0.012 (F=4.46) |
| Base model 2 | Expanded model 2 | ΔSS | p value |
| MCF, age, sex | MCF, age, sex, **SBP** | 0.15 | 0.010 (F=4.59) |

Caption: Univariable linear regression plots (A) showing the relationship between systolic blood pressure and left ventricular ejection fraction (LVEF, left) and myocardial contraction fraction (MCF, right).
A weak positive association was observed between systolic blood pressure and LVEF (R² = 0.018, r = 0.136, β = 0.0513, p < 0.001), while MCF demonstrated a modest inverse association with systolic blood pressure (R² = 0.039, r = -0.199, β = –0.00189, p < 0.001).

Table B demonstrates a multivariable model with β coefficient relationship of SBP with LVEF and MCF as well as nested models demonstrating the model outcomes and delta sum of squares (ΔSS) difference for both LVEF and MCF with the addition of SBP. Nested model comparisons demonstrated that the addition of systolic blood pressure (SBP) contributed significantly to both LVEF and MCF based models, but the magnitude of variance explained differed substantially between them. When SBP was added to the LVEF model (base model 1: LVEF, age, sex), the increase in explained sum of squares was markedly larger (ΔSS = 303, *p* = 0.012; F = 4.46) than when SBP was added to the MCF model (base model 2: MCF, age, sex), where the increase was minimal (ΔSS = 0.15, *p* = 0.010; F = 4.59). This suggests that SBP shares considerably more variance with LVEF than with MCF. In other words, MCF appears to be less influenced by SBP than LVEF is, reinforcing the concept that MCF is a more preload- and afterload-independent measure of myocardial function. This strengthens the rationale for using MCF as a more intrinsic measure of myocardial contractility in prognostic modelling. However, further studies are needed to elaborate on the relationship between MCF and afterload/preload.

S6. Test–retest precision of cardiac MRI parameters comparing machine learning-derived and human-derived measurements.

| Parameter | Test-retest Precision (ML vs Human) | p-value |
| --- | --- | --- |
| EDV | 12.0 (11.2 -12.8) | P <0.01 |
| ESV | 9.1 (8.6 -9.6) | P <0.01 |
| EF | 4.1 (3.9- 4.3) | P <0.01 |
| SV | 9.9 (9.3 -0.6) | P = 0.73 |
| LVM | 15.6 (14.8-16.3) | P <0.01 |
| MCF | 12.5 (11.5-13.5) | P < 0.01 |

Caption: Values represent the test–retest precision (mean difference with 95% confidence intervals). P-values assess the significance of difference between modalities. Stroke volume (SV) showed no significant difference (p = 0.73), whereas all other parameters demonstrated statistically significant differences (p <0.01).
